# Effect of Ionizing Radiation on Human EA.hy926 Endothelial Cells under Inflammatory Conditions and Their Interactions with A549 Tumour Cells

**DOI:** 10.1155/2019/9645481

**Published:** 2019-09-02

**Authors:** Sabine Schröder, Simone Broese, Jana Baake, Dajana Juerß, Stephan Kriesen, Guido Hildebrandt, Katrin Manda

**Affiliations:** Department of Radiotherapy and Radiation Oncology, University Medical Center Rostock, Südring 75, 18059 Rostock, Germany

## Abstract

**Purpose:**

Most tumours are characterized by an inflammatory microenvironment, and correlations between inflammation and cancer progression have been shown. Endothelial cells (ECs), as part of the tumour microenvironment, play a crucial role in inflammatory processes as well as in angiogenesis and could be critical targets of cancer therapy like irradiation. Therefore, in the present study we investigated the effect of ionizing radiation on endothelial cells under inflammatory conditions and their interactions with tumour cells.

**Methods:**

Nonactivated and TNF-*α* treatment-activated human EC EA.hy926 were irradiated with doses between 0.1 Gy and 6 Gy with a linear accelerator. Using a multiplex assay, the accumulation of various chemokines (IL-8, MCP-1, E-selectin, and P-selectin) and soluble adhesion molecules (sICAM-1 and VCAM-1) as well as protein values of the vascular endothelial growth factor (VEGF) was measured in the supernatant at different time points. The adhesion capability of irradiated and nonirradiated A549 tumour cells to EA.hy926 cells was measured using flow cytometry, and the migration of tumour cells was investigated with a scratch motility assay.

**Results:**

In contrast to unirradiated cells, IR of ECs resulted in a modified release of chemokines IL-8 and MCP-1 as well as the adhesion molecules sICAM-1 and VCAM-1 in the EC, whereas concentrations of E-selectin and P-selectin as well as VEGF were not influenced. IR always affected the adhesion capability of tumour cells to ECs with the effect dependent on the IR-treated cell type. TNF-*α* treatment generally increased adhesion ability of the tumour cells. Tumour cell migration was clearly inhibited after IR. This inhibitory effect was eliminated for radiation doses from 0.5 to 2 Gy when, additionally, an inflammatory environment was predominant.

**Conclusions:**

Our results support past findings suggesting that ECs, as part of the inflammatory microenvironment of tumours, are important regulators of the actual tumour response to radiation therapy.

## 1. Introduction

Most tumours are characterized by an inflammatory microenvironment with migration of leukocytes and the release of cytokines and other inflammatory markers [1–4]. Further inflammation-related cells like monocytes are recruited by the secreted cytokines, which in turn release further proinflammatory cytokines and chemokines and, thus, intensify the inflammation. This also creates an inflammatory microenvironment in tumours, which, however, does not originate in an inflammation. This mechanism is referred to as “cancer-related inflammation” [5].

The distinct correlations between inflammation and cancer progression are known. An increased presence of inflammatory cells and soluble inflammatory markers in a primary tumour is associated with a poor prognosis, e.g., due to metastasis [6, 7]. An inflammatory milieu in tumours increases the risk of the development of metastases. For example, the activation of NF-*κ*B could be associated with the metastasis of prostate cancer [8]. High concentrations of monocyte chemoattractant protein-1 (MCP-1), and a proinflammatory chemokine, could also be associated with an increased incidence of metastases in breast, colon, prostate, and neck tumours [9]. Meanwhile, it is recognized that inflammatory markers are involved in tumour progression through the contribution to several mechanisms. Besides the recruitment of inflammatory cells and immune suppression, angiogenesis and metastasis are also among these mechanisms [9].

Endothelial cells (ECs) as part of the inflammatory tumour microenvironment play a critical role in the inflammatory processes. ECs express a variety of cytokines and growth factors, and they are able to recruit leukocytes from peripheral blood [10]. This leukocyte recruitment, mainly of myeloid-derived cells, facilitates tumour survival. It helps to build up a microenvironment with an immune-suppressive characteristic, which prevents the recruitment of tumour-attacking cells and induces a functional nonresponsiveness [9]. Consequently, these mechanisms lead to a tumour-protective environment. Furthermore, ECs are key cells of the walls of blood vessels and exist in all levels of the vascular trees. That makes them essential for blood vessel functions. In normal tissues, microvascular ECs are located very close to the epithelial cells. Therefore, both cell types can communicate with each other by the release of markers like growth factors and hormones [11]. Additionally, epithelial cells are able to get oxygen and nutrients from blood vessels. The same interaction exists in the endothelial-dependent growth of tumour cells [12]. Tumour cells grow very close to ECs in blood vessels. They secrete endothelial mitogens and chemotactic factors, such as the vascular endothelial growth factor (VEGF). This stimulates the proliferation of ECs and the growth of new blood vessels with the help of ECs (angiogenesis) [11]. In return, ECs protect tumour cells by releasing quite a number of growth and survival factors like interleukin-6 (IL-6) [13]. Franses and his coworkers described the potential of ECs to regulate tumour cell function, invasiveness, and response to and elaboration of inflammatory mediators *in vitro* [14]. The growth and expansion of tumours depend on new blood vessels formed by proliferating ECs. As a result of their elevated metabolism, growing tumours have increased oxygen requirement [15]. Therefore, angiogenesis, as the outgrowth of new blood vessels from existing capillaries, is one of the hallmarks of cancer, because without angiogenesis, most solid tumours would not be able to grow more than a few mm^3^ [16]. Consequently, in tumours, besides the secretion of inflammatory markers, ECs also have a crucial role in angiogenesis [17]. As a result, several investigations have been aimed at designing antiangiogenic strategies that target the functions of tumour ECs as the key players in the angiogenic processes [18, 19].

About half of the tumour patients receive radiation treatment during their therapy. Owing to their capability to regulate tumour cell functions like proliferation, invasiveness, and response to and elaboration of inflammatory mediators as well as tumour outgrowth, angiogenesis, and metastasis, ECs may be critical targets of response to tumour therapy like irradiation (IR). Recent studies have clearly demonstrated that IR affects ECs not only after high doses but also after low doses of radiation exposure. ECs are considered important regulators of tumour response after high radiation doses [19]. For moderate and higher radiation doses, a lot of studies have been done to investigate the radiation effect on endothelial cells *in vitro* and a few studies have verified parts of these observations *in vivo* (summarized in [20]). ^137^Cs IR with 20 Gy changed cell morphology in rat lung microvessels; ECs have been observed with the help of scanning electron microscopy [21]. It was demonstrated that high IR doses cause substantial damage of the EC membrane, resulting in hydrolyzation by the enzyme sphingomyelinase, leading, finally, to ceramide release, a signal for apoptosis [19, 22–26]. Irradiated with high doses, after membrane damage, ECs undergo ceramide-mediated apoptosis [27, 28]. It has been stated that tumour ECs undergo radiation-induced apoptosis faster (<6–20 h after IR) than most other tumour cell lines [19]. Apoptosis of EC and microvascular collapse were reported to contribute to the response to IR of transplanted melanoma and fibrosarcoma cell lines [26]. Further studies showed that IR of high doses (8–20 Gy) may target firstly ECs, leading to a subsequent tumour clonogenic cell death [29].

In a publication by Rombouts et al., it was demonstrated that acute low doses of X-rays induce DNA damage and apoptosis in endothelial cells after exposure to 0.1, 0.5, and 5 Gy (X-ray) [30], especially 48 h after exposure with 0.1 and 0.5 Gy, but also, a drop of apoptosis was observed 72 h after exposure in EA.hy926 cells (0.1 and 0.5 Gy). In EA.hy926 cells, irradiation with 5 Gy increased the number of nonapoptotic dead cells at all time points tested. Pluder et al. demonstrated that the apoptotic fraction in EA.hy926 cells was not affected after irradiation of the cells with 0.2 Gy, as well as cellular growth [31]. Another study pointed out that LD-RT included the expression of XIAP (X-linked inhibitor of apoptosis protein) in a discontinuous manner; the maximum was reached after exposure to 0.5 Gy and 3 Gy and a discontinuous apoptosis induction as well as caspase 3/7 activity [32]. A summary of the current knowledge on endothelial cell activation and dysfunction resulting from exposure to ionizing radiation was recently published by Baselet et al. and also pointed out the lack of knowledge regarding the effects of radiation-induced apoptosis in endothelial cells at lower doses [33].

It is known that irradiation of the endothelium and its related proinflammatory signalling cascades result in micro- and macrovascular effects [34], which may also influence tumour growth.

Moreover, IR with low doses affects EC by modulating inflammatory reactions [35, 36]. A great number of studies have investigated the radiation effects on ECs and their role in the anti-inflammatory cascade for the low-dose area (summarized in [37]). Oxidative stress is known to be involved in the disturbance of EC, which may result in further damage of the vascular system [38, 39]. Proteome analyses of EA.hy926 at 4 hours and 24 hours after IR with 0.2 Gy showed alterations in the signalling pathways Ran and RhoA, both associated with diverse experimental situations with oxidative stress reactions [31]. In ECs, activated with TNF-*α* as an inflammatory stimulus, a nonlinear expression and activity of compounds of the antioxidative system were seen [40]. On the basis of different ECs (EA.hy926 and HMVEC), the authors showed a discontinuous expression and enzymatic activity of glutathione peroxidase accompanied by a lowered expression and DNA-binding activity of the transcription factor, nuclear factor E2-related factor 2 (Nrf2). IR of the same two ECs (EA.hy926 and HMVEC) with 0.01 Gy to 2 Gy caused nonlinear dose-dependent effects on the proinflammatory cytokine secretion of IL-8, G-CSF, and PDGF-BB [36]. The mRNA expression levels of those cytokines were nonlinear and dose-dependent and differed in respect of the protein level in the culture supernatant. After exposure to low-dose IR, the nuclear factor kappa B (NF-*κ*B), as one of the most important regulators of proinflammatory gene expression, represented a biphasic activation; a discontinuous expression of the X chromosome-linked inhibitor of apoptosis (XIAP) was also demonstrated [32, 41]. The enhanced expression of anti-inflammatory TGF-*β*1, a decreased E-selectin expression, and a significantly reduced adhesion of leukocytes to ECs after exposure to LD-IR are also a few of the observed effects in the range of 0.3 Gy to 0.7 Gy [42–44]. Besides, the adhesion of Peripheral Blood Mononuclear Cells (PBMCs) to ECs was reduced after low doses of IR [41, 42]. The selected examples clearly show EC as a key player in “cancer-related inflammation,” which regulates diverse aspects of cancer cell function, and may play a crucial role in the radiation response of tumours. But so far, the knowledge about ECs as the targets of IR and the underlying radiobiological mechanisms are limited. Therefore, the objective of this study was to contribute to the filling of this gap by investigating the effect of IR on ECs under inflammatory conditions and their interactions with tumour cells.

## 2. Materials and Methods

### 2.1. Cell Culture

The experiments were performed with human adenocarcinoma epithelial cells A549 and EA.hy926 cells. The EA.hy926 cells, derived from the fusion of human umbilical vein endothelial cells (HUVECs) with a thioguanine-resistant clone of A549, were used as the permanent human endothelial cell line. Both cell lines were obtained from the American Type Culture Collection (ATCC, Manassas, VA, USA). EA.hy926 cells were cultured in Dulbecco's Modified Eagle's Medium (DMEM, Lonza, Cologne, Germany) supplemented with 10% fetal bovine serum (FBS, Merck Millipore, Darmstadt, Germany), 2% sodium pyruvate, 100 U/mL penicillin, and 100 *μ*L/mL streptomycin (Sigma-Aldrich, Hamburg, Germany) in 75 cm^2^ flasks at 37°C and 5% CO_2_. A549 were cultured in DMEM supplemented with 10% FBS, 100 U/mL penicillin, and 100 *μ*L/mL streptomycin in 75 cm^2^ flasks at 37°C and 5% CO_2_.

### 2.2. TNF-*α* Stimulation

The cells were supplemented with TNF-*α* (R&D Systems, Wiesbaden, Germany) before IR to simulate an inflammatory environment and stimulate the secretion of inflammatory markers or adhesion molecules. Therefore, the cell medium was replaced 24 hours after seeding by serum-free medium with or without supplementation of 10 ng/mL TNF-*α* 2 hours before IR.

### 2.3. Ionizing Radiation (IR)

Cells were irradiated 26 hours after seeding at room temperature utilizing a Siemens ONCOR Expression linear accelerator (Siemens, Erlangen, Germany) with a dose rate used in clinical applications of 3.75 Gy/min (photons 6 MV). The irradiation setup ensures a homogeneous dose distribution within the whole cell culture flasks. Cells were transported in their respective cell culture dishes in a sterile box to the linear accelerator and were placed on the disinfected irradiation table and irradiated directly from below. The irradiation was carried out with the following doses: 0.1 Gy, 0.5 Gy, 2 Gy, 4 Gy, and 6 Gy. Sham-irradiated samples (0 Gy) were kept at room temperature in the control room during irradiation.

### 2.4. Analysis of Cellular Metabolic Activity

The water-soluble tetrazolium 1 (WST-1) assay (Roche Deutschland GmbH, Mannheim, Germany) was used to detect the metabolic activity of the cells 2 hours, 24 hours, and 48 hours after IR. Therefore, cells were seeded in a density of 1,000 cells/well in triplicates in 96-well plates, grown under standard conditions, and activated with TNF-*α* or were not activated. WST-1 was added 2 hours before the spectrophotometric measurement following the assay's specific instruction for each respective time point. The assay is based on the cleavage of tetrazolium salts to formazan by cellular enzymes. An expansion in the number of viable cells results in an increase in the overall activity of mitochondrial dehydrogenases, and the formation of formazan is directly proportional to the number of metabolic active cells in the culture. The spectrophotometric measurement was performed with the ANTHOS Zenyth 340r reader (Anthos Mikrosysteme GmbH, Krefeld, Germany).

### 2.5. Analysis of Cellular Vitality

Cells were seeded in a density of 5 × 10^4^ cells/well in duplicates in 24-well plates, grown under standard conditions, and activated 2 hours before IR with TNF-*α* or were not activated. At 2 hours, 24 hours, 48 hours, and 72 hours after IR cells from supernatants were collected, cells in wells were trypsinized with 300 *μ*L of 0.25% trypsin-EDTA (Merck KGaA, Darmstadt, Germany) and also collected. Measurement of the live/dead ratio of both cell fractions (supernatant and cells in wells) was performed using the CASY 150 *μ*M Cell Counter and Analyzer (OLS-OMNI Life Science GmbH & Co. KG, Bremen, Germany). For that, 200 *μ*L of each cell suspension was diluted with 10 mL CASYton (OLS-OMNI Life Science GmbH & Co. KG) prior to automated cell counting.

### 2.6. Analysis of Various Proteins

#### 2.6.1. Sample Collection for Protein Measurement

Cells were seeded in a density of 5 × 10^4^ cells/well in 24-well plates and cultivated to confluence for 24 hours under standard conditions. The medium was replaced by serum-free medium with or without TNF-*α* 2 hours before IR. Subsequently, cells were exposed to radiation. Supernatants were collected 2 hours, 5 hours, 24 hours, 48 hours, and 72 hours after irradiation procedure and stored at -80°C until further measurement.

#### 2.6.2. Measurement of Accumulated Proteins

Accumulated levels of IL-8, MCP-1, E-selectin, P-selectin, sICAM-1, VCAM-1, and VEGF were quantified in supernatants harvested from EC by using a Luminex® Magnetic Screening Assay from R&D Systems (Minneapolis, MN, USA) according to the manufacturer's protocol. Data were acquired using the Bio-Plex® 200 suspension array system and analysed with the Bio-Plex Manager™ Software (version 4.1).

### 2.7. Cell Adhesion Assay

#### 2.7.1. Cell Seeding and CFSE Staining

EA.hy926 cells were seeded in duplicates in 6-well plates 26 hours before IR to reach a confluent cell monolayer. A549 tumour cells were then stained with CellTrace™ CFSE (cat. no. C34554, Molecular Probes, Thermo Fisher Scientific, Darmstadt, Germany) in a concentration of 1.2 mM for 10 minutes, washed twice with PBS, and seeded in a T75 flask with a density of 5 × 10^5^ cells/flask for 24 hours. The dye crosses the plasma membrane and covalently binds to intracellular amines on the surface. Thus, when the labelled cells divides, its progeny are endowed with 50% of carboxyfluorescein-tagged molecules and each cell division is assessable by measuring the respective decrease in fluorescence.

#### 2.7.2. Irradiation

The medium was replaced with serum-free medium 2 hours before IR, and EA.hy926 cells were stimulated with TNF-*α* or remained unstimulated. Three different radiation approaches were examined: IR of only EA.hy926 or A549 and IR of both cell lines at the same time under the same conditions on the same irradiation table. After exposure to IR, the cells were kept in the incubator for an additional 24 hours.

#### 2.7.3. Adhesion and Flow Cytometer Analysis

Tumour cells were counted 24 hours after IR, and 5 × 10^5^ cells/well were added to EA.hy926 for 2 hours in the incubator under static conditions. In order to eliminate nonbound A549, the wells were carefully rinsed four times with phosphate-buffered saline (PBS, PAA Laboratories GmbH, Cölbe, Germany) followed by trypsin treatment according to the cell culture standard protocol (5 mL of trypsin added to the cells, kept in the incubator for 5 to 7 minutes) to detach the cells and flow cytometric analysis. The flow cytometer Cytomics FC 500 (Beckman Coulter GmbH, Krefeld, Germany) with an argon laser and an excitation wavelength of 488 nm was used for the experiments. Data acquisition and analysis was performed by CXP Analysis software provided with the FC 500. The adhesion of unirradiated A549 to the unirradiated and nonstimulated EA.hy926 was set to 1 and is referred to in this study as the control.

### 2.8. Scratch Motility Assay

The ability of A549 cells to migrate into a cell-free area after IR and/or TNF-*α* treatment was examined via scratch assay. Therefore, cells were seeded in duplicates in a 4-well chamber slide with a density of 1 × 10^4^ cells/well (Baacklab, Schwerin, Germany) for 48 hours to reach confluency. The medium was replaced 2 hours before IR with serum-reduced medium containing 0.5% FBS, to minimize proliferation of the cells, and supplemented with TNF-*α* or remained unstimulated. Immediately after exposure to IR, a scratch with a 10 *μ*L pipette tip was created, the debris was removed, and cells were kept in a serum-reduced medium containing 0.5% FBS. Pictures of the cell-free area were taken directly after the scratch as well as after 24 hours and 48 hours. Markings close to the scratch were generated, so the same fields were observed during image acquisition. Pictures were taken with a Nikon Eclipse TE300 Microscope (Nikon, Düsseldorf, Germany), and the scratch was analysed using the public domain software ImageJ (National Institutes of Health). Wound size was quantified as the ratio of the cell-free area to the area of the initial wound (mean percentage) for each time point.

### 2.9. Statistical Analysis

All data are presented as means ± standard deviation (SD) on the basis of at least three to four independently performed experiments. The statistical significance of differences was assessed by Student's *t*-test. A value of *p* < 0.05 was considered as statistically significant.

## 3. Results

### 3.1. Influence of IR on Cellular Metabolic Activity and Cellular Vitality

The influence of IR on metabolic activity was investigated to exclude an alteration of metabolic activity as a reason of the changes in subsequent analyses. The cellular metabolic activity was measured in EA.hy926 and A459 cells 2 hours, 24 hours, and 48 hours after different doses of IR. The metabolic activity slightly increased in EC EA.hy926 over time but remained unaffected by radiation treatment or activation of the cells with TNF-*α* prior to IR (Supplement [Supplementary-material supplementary-material-1]). In A459 tumour cells, the time-dependent increase was more distinct compared to ECs. Furthermore, metabolic activity increased with TNF-*α* activation but was likewise unaffected by radiation treatment (Supplement [Supplementary-material supplementary-material-1]). To exclude also the influence of the ratio of living and dead cells as a reason of the changes in subsequent analyses, additionally, cellular vitality of EC EA.hy926 cells 2 hours, 24 hours, 48 hours, and 72 hours after different doses of IR was measured (Supplement [Supplementary-material supplementary-material-1]). It was demonstrated that, with one exception, no significant alterations in the live/dead ratio of ECs after IR were induced in general. All subsequently observed changes in protein secretion, adhesion, and cell motility resulted due to IR not due to alteration of metabolic activity or alterations in the live/dead ratio of cells.

### 3.2. Effect of IR on Protein Accumulation in the Culture Medium

The influence of irradiation on cytokine secretion by EC, with and without simulating an inflammatory environment (±TNF-*α*), was analysed. Therefore, concentrations of different chemokines like interleukin-8 (IL-8), monocyte chemoattractant protein-1 (MCP-1), E-selectin, and P-selectin were measured after IR. Furthermore, the concentration of accumulated soluble intercellular adhesion molecule-1 (sICAM-1) and vascular cell adhesion molecule-1 (VCAM-1) was investigated, and the vascular endothelial growth factor (VEGF) in the cell culture medium was analysed.

#### 3.2.1. Effect of IR on Release of IL-8

**Figure 1:**
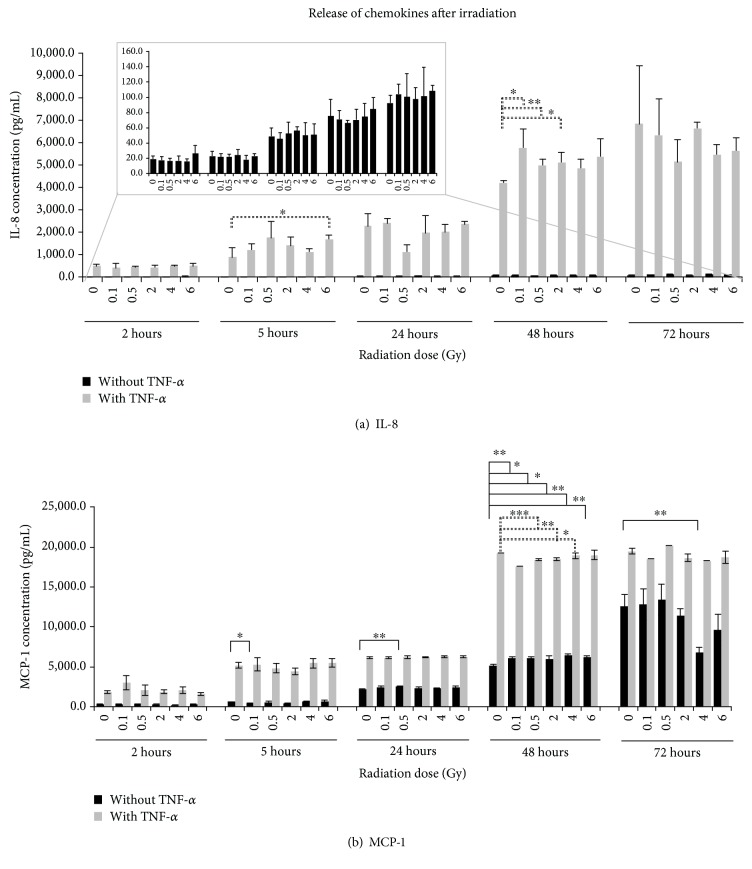
Accumulated levels of (a) interleukin-8 (IL-8) and (b) monocyte chemoattractant protein-1 (MCP-1) in the supernatant of EA.hy926. The protein concentration was determined by multiplex assay at five time points after irradiation with photons. Changes in protein concentrations are presented as mean (pg/mL) ± standard deviation (SD) from three independent experiments; asterisks illustrate significance: ^∗^*p* < 0.05, ^∗∗^*p* < 0.01, and ^∗∗∗^*p* < 0.001.

Interleukin-8 is a cytokine with proangiogenetic and antiapoptotic effects on a variety of cells like monocytes or ECs and promotes the migration of ECs into the ECM. The accumulation of IL-8, derived from nonactivated EA.hy926 cells, was observed in a time-dependent manner from 2 hours to 72 hours after IR with a maximum concentration of approximately 100 pg/mL IL-8 at the latest time point (Figure 1(a)). The TNF-*α* stimulation of the EC resulted in a time-dependent massive increase of IL-8 concentration in the supernatant with a significant increase 5 hours after IR with 6 Gy (1.9-fold) and 48 hours after IR with 0.1 Gy, 0.5 Gy, and 2 Gy compared to sham-irradiated samples. The IL-8 accumulation was not linear and dose-dependent at all the tested time points with an average maximum concentration of 6,840 pg/mL IL-8 72 hours after IR.

#### 3.2.2. Effect of IR on Release of MCP-1

MCP-1, which induces a chemotactic activity in several cell types, can promote the infiltration of monocytes and macrophages into various tumours as well as enhance the proliferation and promote the migration and infiltration of tumour cells. The accumulation of MCP-1 derived from EA.hy926 without TNF-*α* treatment before IR was also observed in a time-dependent manner from 2 hours to 72 hours after IR with a concentration ranging from 291 pg/mL to 11,103 pg/mL (Figure 1(b)). Significant dose-dependent changes in accumulation were detected 5 hours (0.1 Gy) and 24 hours (0.5 Gy) after IR as well as 48 hours at all tested doses and 72 hours (4 Gy) after IR compared to 0 Gy samples. The activation of the cells with TNF-*α* also resulted in a time-dependent increase from 2 hours to 5 hours after IR and a massive increase from 24 hours to 48 hours but with higher concentrations compared to nonstimulated samples. No remarkable changes in the accumulation of MCP-1 were observed between 5 and 24 hours as well as 48 and 72 hours postirradiation.

The concentrations ranged from approximately 2,064 pg/mL to 18,973 pg/mL. The level of accumulated MCP-1 in the supernatant remained similar 72 hours after IR compared to samples at 48 hours. Dose-dependent decreases in MCP-1 were detected 48 hours after IR at all tested doses compared to the 0 Gy sample at the respective time points, but only for doses of 0.1 Gy, 0.5 Gy, 2 Gy, and 4 Gy, the decrease was statistically significant.

#### 3.2.3. Effect of IR on Release of E- and P-Selectins

Selectins not only mediate the rolling of leukocytes on the surface of endothelial cells but are also involved in tumour metastasis. The accumulated levels of E- and P-selectins did not change over the examined time period of 2 hours to 72 hours after IR (See supplement Fig. [Supplementary-material supplementary-material-1] and [Supplementary-material supplementary-material-1]). The activation of the EC via TNF-*α* before IR did not result in an altered or higher concentration of E- and P-selectins. Moreover, no significant influence on the secretion of the two selectins was observed after IR.

#### 3.2.4. Effect of IR on Release of sICAM-1

**Figure 2:**
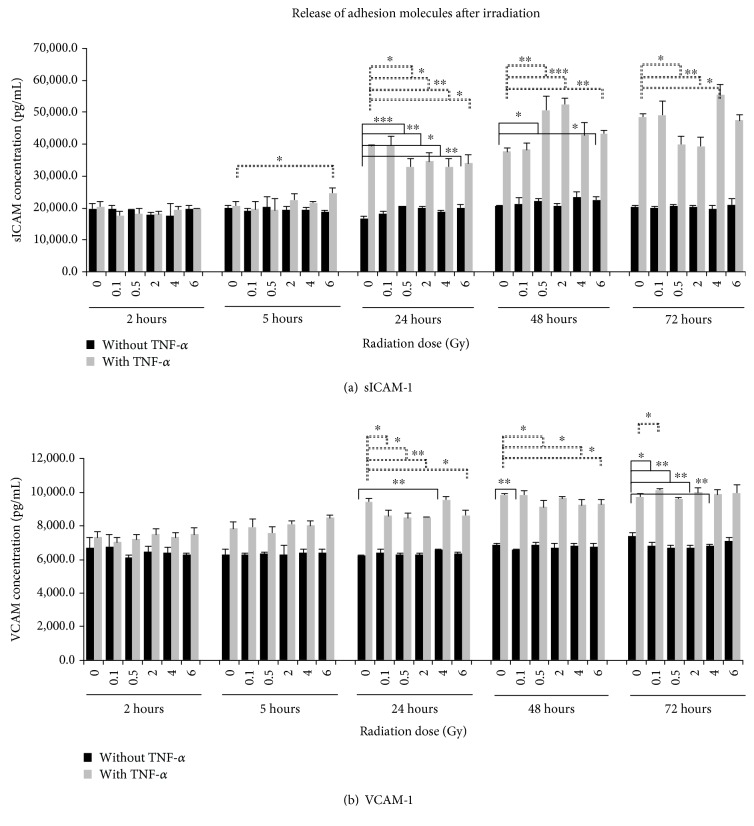
Accumulated levels of (a) soluble intercellular adhesion molecule-1 (sICAM-1) and (b) vascular cell adhesion molecule-1 (VCAM-1) in the supernatant of EA.hy926. The protein concentration was determined by multiplex assay at five time points after irradiation with photons. Changes in protein concentrations are presented as mean (pg/mL) ± standard deviation (SD) from three independent experiments; asterisks illustrate significance: ^∗^*p* < 0.05, ^∗∗^*p* < 0.01, and ^∗∗∗^*p* < 0.001.

ICAM-1 is a cell surface glycoprotein typically expressed on ECs and plays a key role during inflammation and immune responses as well as transendothelial migration. The accumulation of sICAM-1 derived from nonactivated EA.hy926 remained at the same level over a time period of 2 hours to 72 hours after IR with concentrations ranging from 18,982 pg/mL to 21,768 pg/mL (Figure 2(a)). Significant higher concentrations of sICAM-1 could be measured 24 hours after IR with 0.5 Gy up to 6 Gy and 48 hours after IR with doses of 0.5 Gy and 6 Gy compared to the nonirradiated samples for each time point. The stimulation of the EC with TNF-*α* before IR resulted in clearly higher sICAM-1 concentrations at time points 24 hours to 72 hours after IR. Significantly decreased sICAM-1 levels were measured 24 hours after IR with 0.5 Gy to 6 Gy and 72 hours after IR with 0.5 Gy and 2 Gy but significantly higher levels 48 hours after IR with 0.5 Gy, 2 Gy, and 6 Gy as well as 72 hours after IR with 4 Gy compared to the 0 Gy control for each time point. The sICAM-1 concentrations in stimulated ECs ranged on an average from 18,913 pg/mL up to 46,639 pg/mL.

#### 3.2.5. Effect of IR on Release of sVCAM-1

VCAM-1 not only mediates the adhesion of a variety of cells to the endothelium but also takes part in the regulation of immune surveillance and inflammation, besides other adhesion molecules. The accumulation of sVCAM-1 derived from unstimulated ECs on an average ranged from 6,332 pg/mL to 6,933 pg/mL (Figure 2(b)). No time-dependent alterations were detectable from 5 hours to 72 hours. In contrast, stimulation of the cells with TNF-*α* led to a distinct time-dependent increase of accumulated soluble VCAM-1 in the culture supernatant at time points from 24 hours or later. Thereafter, dose-dependent changes of sVCAM-1 concentrations could also be seen. Significant lower concentrations were measured 24 hours as well 48 hours after IR, but a significantly higher level was measured 72 hours after IR with 0.1 Gy. On an average, the sVCAM-1 concentrations ranged from 7,318 pg/mL (2 hours) to 9,897 pg/mL (72 hours).

#### 3.2.6. Effect of IR on Release of VEGF

VEGF is a signalling molecule playing a pivotal role in vasculogenesis and angiogenesis with stimulatory properties on EC division and migration. The concentration of accumulated VEGF in the supernatant of irradiated ECs increased time-dependently from 2 hours to 72 hours after IR (see supplement Fig. [Supplementary-material supplementary-material-1]). The secretion was not linear and dose-dependent, but a statistical significance was not detected. After cells were stimulated with TNF-*α* before IR, the measured concentration of VEGF was higher compared to that of nonstimulated samples 24 hours to 72 hours after IR. But in comparison to the control (0 Gy) also, the changed levels were nonlinearly dose-dependently altered.

### 3.3. Effect of IR and Inflammation on Adhesion of Tumour Cells to Endothelial Cells

**Figure 3:**
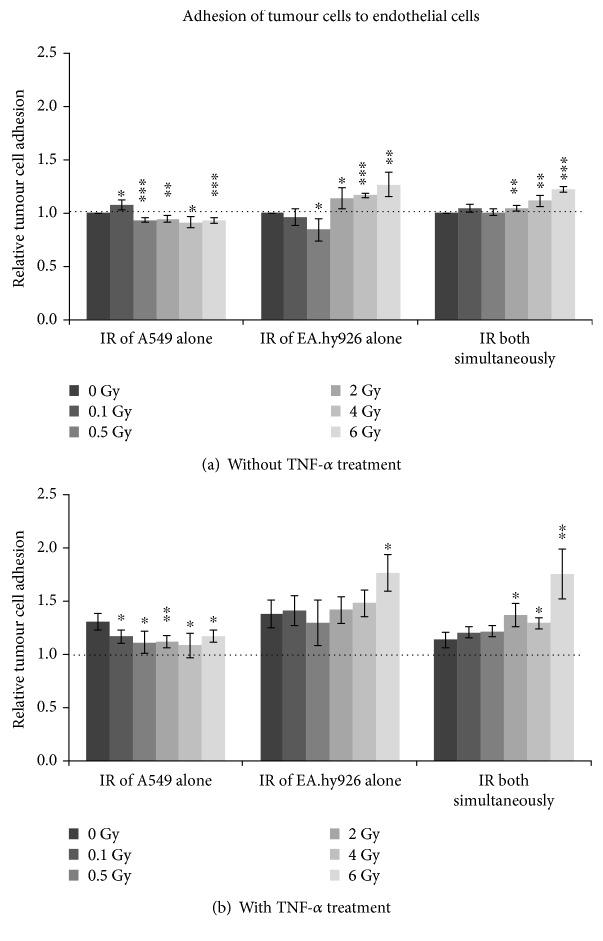
Adhesion of A549 to EA.hy926 (a) without TNF-*α* and (b) with TNF-*α* stimulation of endothelial cells. The tumour cell adhesion was determined by flow cytometry 26 hours after irradiation with photons. Relative cell adhesion is presented as mean ± standard deviation (SD) from four independent experiments; asterisks illustrate significance: ^∗^*p* < 0.05, ^∗∗^*p* < 0.01, and ^∗∗∗^*p* < 0.001.

To quantify the influence of IR on the adhesive capacity of tumour cells, the adhesion of A549 to EA.hy926 was measured under different conditions (Figure 3). Results were normalized to the effect received with untreated cells (0 Gy, no TNF-*α*). The irradiation of A549 alone led to a significant, dose-dependent reduced adhesion of the tumour cells to ECs after 0.5 Gy to 6 Gy (Figure 3(a)). Only after 0.1 Gy was a significant higher adhesion detected compared to the control. When ECs were additionally stimulated with TNF-*α* before IR, the EC adhesiveness for A549 cells was clearly elevated compared to that of nonstimulated ECs. For those samples, at all the tested IR doses, cell adhesion decreased significantly compared to 0 Gy (Figure 3(b)).

When only EA.hy926 cells were irradiated and not activated before IR, the adhesion of A549 to ECs was altered with a significantly lower adhesion after IR with 0.5 Gy. For higher IR doses up to 2 Gy to 6 Gy, adhesion rose significantly and dose-dependently compared to the control (Figure 3(a)). The additional TNF-*α* activation of ECs also led to a dose-dependent increase in the adhesion of A549 cells compared to the control, which was significant after 6 Gy (Figure 3(b)). Cell adhesion in TNF-*α*-simulated samples was generally higher than that in unstimulated cells.

The simultaneous IR exposure of A549 and EA.hy926 cells caused a dose-dependent higher adhesion of tumour cells to ECs, which was significant at doses of 2 Gy to 6 Gy (Figure 3(a)). An inflammatory activation of ECs before IR resulted in the same dose-dependent adhesion enhancement (Figure 3(b)). TNF-*α* stimulation also resulted in generally higher cell adhesion levels compared to unstimulated cells.

### 3.4. Effect of IR and Inflammation on Cell Motility of Tumour Cells (Migration Potential)

**Figure 4:**
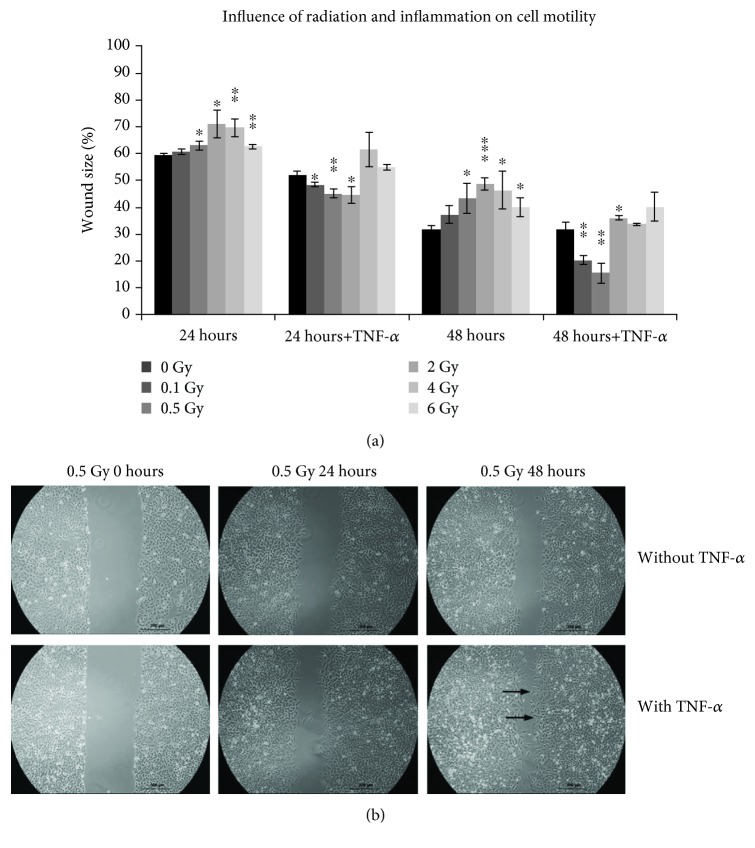
Analysis of tumour cell migration by *in vitro* scratch assay. (a) Quantification of the wounded area. The results show what percentage of the originally free area (100%) was not covered by cells. Relative cell adhesion is presented as mean ± standard deviation (SD) from four independent experiments, obtained in duplicates for each approach; asterisks illustrate significance: ^∗^*p* < 0.05, ^∗∗^*p* < 0.01, and ^∗∗∗^*p* < 0.001. (b) Representative phase contrast photographs of the wounded area taken immediately after the scratch (0 hours) as well as 24 hours and 48 hours after IR, without or with TNF-*α* treatment of the cells. Black arrows point out the altered cell morphology observed in cells 48 hours after irradiation with 0.5 Gy and stimulation with TNF-*α*.

To investigate the effect of IR and inflammation on migration, A549 cells were treated with TNF-*α* at 2 hours before IR exposure or left untreated. The results show the percentage of the originally free area that was not covered by cells. Tumour cells responded to IR with a significant dose-dependent reduced migration into the cell-free gap 24 hours after IR (Figure 4(a)). A reduced migration was also detected 48 hours after IR with the same significant dose-dependent effect compared to nonirradiated samples. If A549 cells were treated with TNF-*α* to create an inflammatory environment, the migration into the cell-free gap was significantly enhanced in a dose-dependent manner from 0.1 Gy to 2 Gy but decreased at the higher doses of 4 Gy and 6 Gy (not significant). The enhancement of tumour cell migration reached a significant maximum 48 hours after IR under TNF-*α* treatment after a dose of 0.5 Gy (*p* < 0.01); at doses of 2 Gy to 6 Gy, the migration of A549 was dose-dependently inhibited compared to 0 Gy. In addition, in samples with TNF-*α*, an altered cell morphology could be observed with an increase in elongated cells with pseudopodia, indicating an epithelial-mesenchymal transition (EMT; Figure 4(b)).

## 4. Discussion

ECs, as key players in “cancer-related inflammation,” regulate diverse aspects of cancer cell functions. Besides the secretion of inflammatory markers, ECs also have a crucial role in angiogenesis and may play a crucial role in the radiation response of the actual tumour. The objective of this study was to investigate the effect of IR on ECs under inflammatory conditions and their interactions with tumour cells in more detail. Therefore, nonactivated and activated human EC EA.hy926 were irradiated with doses between 0.1 Gy and 6 Gy with a linear accelerator and secretion of cytokines, adhesion of A549 tumour cells to the EC and the cell motility of tumour cells were characterized.

Owing to the results derived from the analyses of metabolic activities, an alteration in metabolic activity as a cause of the observed changes in subsequent analyses—secretion of cytokines, adhesion of tumour cells to ECs, and cell motility of tumour cells—could be excluded. It was demonstrated that IR up to single doses of 6 Gy and until 48 hours after IR did not affect the metabolic activity significantly. Moreover, no significant changes were detected in an inflammatory environment after activation with TNF-*α*. The results verified the preserved metabolic activity of the tumour as well as the EC after IR. The increase of metabolic activity was solely explained by cell growth over the observed time frame and, therefore, the gain of viable cells metabolizing the WST-1. In former studies, too, we observed no significant loss of cell viability in three murine ECs—mlEND1, H5V, and bEND3—caused by IR in the investigated dose range [35]. The analysis of metabolic activity was used by many other authors, e.g., Cervelli and coworkers, who irradiated human umbilical vein endothelial cells (HUVECs) with X-rays and observed no significant loss of cell viability caused by IR [45].

One main goal of this study was to analyse the cytokine secretion of the EC activated with TNF-*α* before IR for the simulation of an inflammatory situation. The tumour necrosis factor *α* (TNF-*α*) is one of the important proinflammatory cytokines involved in the tumour microenvironment [3]. Therefore, all experimental setups in this study were investigated with cells stimulated with TNF-*α* before treatment and compared with nonstimulated cells. Following the release of various chemokines (IL-8, MCP-1, E-selectin, and P-selectin) and adhesion molecules (sICAM-1 and sVCAM-1), the protein values of the vascular endothelial growth factor (VEGF) were measured in the supernatant at different time points after IR.

Interleukin-8 is a cytokine with a proangiogenetic and antiapoptotic effect on a variety of cells like monocytes or ECs and promotes the migration of ECs into the extracellular matrix. The angiogenic property of IL-8 and other CXC chemokines was investigated for many years [46]. IL-8 promotes an angiogenic response in ECs and the recruitment of neutrophils to the tumour site via its paracrine activity [47]. In the present study, the secretion of IL-8 through ECs was investigated over 72 hours after IR and with or without stimulation by TNF-*α* before IR. In activated and nonactivated EA.hy926 cells, IL-8 secretion rose over time 2 hours to 72 hours. The TNF-*α* stimulation resulted in a massive increase of IL-8 concentration in the supernatant. The IL-8 release was nonlinear and dose-dependent at all the tested time points.

In experiments published by Van der Meeren et al., HUVECs secreted IL-8 time-dependently over 6 days, which is in accordance with our findings measured over 3 days as well as the higher secretion, if cells were treated with TNF-*α* before IR [48]. When the cells were irradiated with 10 Gy, the IL-8 concentration measured 6 days post-IR was 136-fold higher compared to nontreated samples, showing a massive increase of IL-8 concentration in the supernatant similar to our observations.

High IL-8 concentrations are known to predominate in human malignant melanomas [9, 49]. When high levels of the IL-8 receptor CXCR2 are present in melanomas, an enhanced metastatic potential could be determined [50]. But after neutralization of IL-8, the stimulation of the metastatic outgrowth could be weakened [51].

Monocyte chemoattractant protein-1 (MCP-1) can promote the infiltration of monocytes and macrophages into various tumours as well as enhance the proliferation and promote the migration and infiltration of tumour cells. High concentrations of MCP-1 could also be associated with an increased incidence of metastases in breast, colon, prostate, and neck tumours [9]. MCP-1 secretion of EA.hy926 cells increased over the observation time from 2 hours to 72 hours independently of cell activation. As verified for IL-8, a TNF-*α* activation of the cells resulted in a massive increase in MCP-1 secretion, too. Moreover, the MCP-1 release was nonlinear and dose-dependent.

An increase in the proinflammatory marker, MCP-1, by HUVECs after stimulation with a higher concentration of TNF-*α* (20 ng/mL) was described by Gerhardt et al. [52]. We could achieve this effect with a 50% lower concentration (10 ng/mL).

It has also been stated that microvascular endothelial cells from human lungs (HLEC) can secrete chemokines like MCP-1 or IL-8 generally and in higher concentrations after TNF-*α* stimulation [53]. In our former study in murine EC mlEND.1, the concentration of MCP-1 48 hours after IR with 0.1 Gy was 10-fold (2D) or 5.4-fold (3D) higher in activated cells compared to the nonactivated cells [35].

Well known as mediators of cell-cell and cell-matrix interactions and essential for a variety of processes including tumour development, invasion, and metastasis are adhesion molecules. Several studies focused on the effect of IR on different members of adhesion molecules, like immunoglobulins and selectins; and their modulation by IR seems to play a role in radiation-induced tumour response, tumour inflammation, and metastasis and angiogenesis [54]. Therefore, in the present study, levels of sE-selectin, P-selectin, soluble ICAM, and VCAM, secreted by ECs, were examined after IR and/or the influence of a proinflammatory stimulus over a time course of 72 hours was ascertained.

Whereas no altered concentrations of E- and P-selectins could be observed, both cell adhesion molecules (CAMs) were released in the supernatant in higher concentrations after stimulation with TNF-*α*, where the enhanced secretion was more distinct for sICAM. Significant dose-dependent changes in the concentration were also detected for 4 out of 5 time points (sICAM) or 3 out of 5 time points (VCAM).

IR-induced expressions of leukocyte adhesion molecules like ICAM and VCAM by ECs were also described in other studies [54–57]. Direct and indirect mechanisms for the enhanced expression of adhesion molecules after IR were suggested and summarized by Baluna et al. [54]. As a result, this IR-induced expression finally led to an increased leukocyte extravasation from a circular flow into the tumour tissue [16].

Our experiments indicate that the exposure to various doses of IR did not alter the secretion of E- and P-selectins. A time-dependent increase or decrease of these adhesion molecules could not also be detected. The activation of EC with 10 ng/mL TNF-*α* did not result in any increase of the two molecules compared to nonactivated samples. This effect was also seen in a previous study by Galley et al., where the exposure to TNF-*α* for 24 hours also resulted in no higher sE-selectin concentrations in the supernatant of EA.hy926 cells [58]. Similar findings were published by Thornhill et al. [59]. The activation of EA.hy926 cells with TNF-*α* resulted in only a slight increase of sE-selectin compared to nonactivated cells. Therefore, we conclude that these two molecules do not play a major role in the adhesion of A549 to EA.hy926 cells in this experimental setup.

Another signalling molecule playing a pivotal role in blood vessel formation and angiogenesis with stimulatory property on ECs is the vascular endothelial growth factor (VEGF). *In vitro* VEGF stimulates EC division and migration. An elevated expression of VEGF is found in a number of tumours. The investigated EC EA.hy926 secreted VEGF, but no dose-response relationship of IR and VEGF secretion could be observed. After cells were activated with TNF-*α* before IR at later time points, the concentration of VEGF was slightly higher compared to that of the nonactivated samples. But also in comparison to the control, the changed levels were not dose-dependently altered. From our results, we assume that like the E- and P-selectins, VEGF secretion from EA.hy926 does not play a major role in the adhesion of A549 to ECs in this experimental setup.

Critical steps involved in haematogenous metastasis of tumour cells are the adhesion to the endothelium, the extravasation through the endothelial layer, and the invasion into the extracellular matrix. In the first two steps, tumour cells interact with endothelial cells in order to invade through the matrix. Therefore, the influence of IR under inflammatory conditions on adhesion of tumour cells to endothelial cells as well as the tumour cell migration was the purpose of our further investigation.

The influence of IR on the adhesion of A549 tumour cells to EA.hy926 cells was measured under different conditions. IR of only A549 cells led to a significant, dose-dependent reduced adhesion of the tumour cells to ECs after 0.5 Gy to 6 Gy. This was in contrast to the effect described by Kiani et al., where A549 cell adhesion to ECs increased after IR. But in their study, HUVECs were used as ECs [60].

In our study, the tumour cell adhesion to ECs was altered only when the EA.hy926 cells were irradiated, with reduced adhesion after low doses of IR (0.1 Gy and 0.5 Gy) and rose dose-dependently for higher IR doses (2 Gy to 6 Gy). It is well known that high doses of IR result in proinflammatory reactions. But low doses can also cause anti-inflammatory reactions [44, 61, 62]. Over the past decades, many studies revealed the anti-inflammatory effect of low-dose IR on various cells with different responses to the applied radiation doses, which were summarized in Rödel et al. [37]. Several studies already revealed the reduced adhesion to ECs, e.g., of Peripheral Blood Mononuclear Cells (PBMCs), after low-dose IR [41, 42]. In a study by Rödel et al., the adhesion of PBMCs to activated EA.hy926 EC cells was significantly reduced 24 hours after IR with 0.5 Gy [41]. The simultaneous IR exposure of A549 and EA.hy926 cells resulted in a dose-dependent higher adhesion of tumour cells to ECs at higher doses of 2 Gy to 6 Gy. Kiani et al. reported no significant effect on adhesion after simultaneous IR of A549 cells and HUVECs [60]. But as mentioned above, different ECs were used. In our earlier studies, we compared the binding of monocytes to three different EC lines (H5V, mlEND1, and bEND3). It has been clearly observed that not only a change in the inflammatory cytokine release but also adhesion capacity like that of monocyte binding to ECs depended on the origin of the ECs [35]. The dependence of IR response on the origin of ECs was also described by Nicolson and his coworkers [21]. In their study, 24 hours after IR, the adhesion of tumour cells to rat lung microvessel ECs increased but not to mouse brain microvessel ECs.

In the present study, when cells were additionally stimulated with TNF-*α* before IR, the tumour cell adhesion to EC clearly increased compared to nonstimulated cells, but the same dose-dependencies were observed. The ability of TNF-*α* to stimulate and activate different cell types like ECs was described by various other researchers and confirmed in numerous experimental setups. O'Carroll and coworkers reported the stimulatory effect of TNF-*α* on a human EC line (human cerebral microvascular EC (hCMVEC)) [63].

Besides the adhesion to the endothelium, further critical steps involved in haematogenous metastasis of tumour cells are the extravasation through the endothelial layer and the invasion into the extracellular matrix. Against this background, the effect of IR and inflammation on A549 tumour cell migration was investigated. Tumour cells responded to IR with a significant dose-dependent reduced migration 24 hours and 48 hours after IR. Interestingly, if A549 cells were in an inflammatory environment, the migration was significantly enhanced at low IR doses but reduced at higher doses. As described above for the tumour cell adhesion, for tumour cell migration, too, proinflammatory reactions at high doses of IR and anti-inflammatory response at low doses of IR could be demonstrated.

In addition, only tumour cells that were activated with TNF-*α*, with modified cell morphology with increased elongated cells with pseudopodia indicating epithelial-mesenchymal transition (EMT), could be observed. This is in agreement with the work of Yamauchi and coworkers, which showed that treatment with TNF-*α* promotes EMT [64]. Shiozaki et al. also reported that the addition of TNF-*α* promotes both EMT and migration in A549 cells [65]. Jung et al. also reported radiation-induced modifications in morphology (longer cell shape and increased occurrence of pseudopodia) and alterations in adhesion and cell motility of A549 cells [66]. But in contrast to our results, instead of reduction, stimulation of the A549 motility after IR with 6 Gy was shown. Maybe different conditions of cultivation and during IR were reasons for the opposite observations. While we used a serum-reduced medium containing 0.5% FBS, Jung and coworkers used 1% FBS. Furthermore, in our study for IR of the cells, a linear accelerator with a dose rate of 3.75 Gy/min was used, whereas Jung et al. irradiated with a caesium source delivering 7.3 Gy/min at room temperature. The migration and invasion of cells during the process of metastasis is related to the EMT as well as increased migration and motility. The investigated epithelial tumour cells, A549, used in our study were derived from a human adenocarcinoma of the lung. Owing to their metastatic nature, lung tumours are characterized by poor survival statistics [66].

## 5. Conclusion

Endothelial cells (EC), as part of the tumour microenvironment, play a critical role in the inflammatory processes of “cancer-related inflammation.” They express a variety of cytokines and growth factors and are essential for blood vessel functions. This importance suggests that ECs are critical targets of response to irradiation during tumour therapy. In our study, we demonstrated that IR of ECs results in the modified release of chemokines (IL-8, MCP-1) as well as adhesion molecules (sICAM-1, sVCAM-1) in the human EC. The adhesion capability of A549 tumour cells to ECs was also affected by IR; the nature of the effect was dependent on the IR-treated cell type. An inflammatory milieu of TNF-*α* treatment generally increased adhesion ability of the tumour cells. Tumour cell migration was clearly inhibited by IR. This inhibitory effect was eliminated in low and moderate radiation doses when an inflammatory environment was predominant. Thus, our results support past findings suggesting that ECs, as part of the inflammatory microenvironment of tumours, are important regulators of the actual tumour response to radiation therapy after low as well as high radiation doses.

## Data Availability

The data supporting this study are provided in Results or as supplementary information accompanying this paper. Further datasets used and/or analysed during the current study are available and are stored by the authors at the University Medical Center Rostock.

## Abbreviations

CAMs: Cell adhesion molecules
DMEM: Dulbecco's modified Eagle's medium
EC: Endothelial cells
ECM: Extracellular matrix
EMT: Epithelial-mesenchymal transition
Gy: Gray
HMVECs: Human microvascular endothelial cells
HUVECs: Human umbilical vein endothelial cells
IL: Interleukin
IR: Irradiation
KC: Keratinocyte-derived chemokine
MCP-1: Monocyte chemoattractant protein-1
NF-*κ*B: Nuclear factor kappa B
PBMCs: Peripheral blood mononuclear cells
PBS: Phosphate-buffered saline
TNF-*α*: Tumour necrosis factor alpha
VEGF: Vascular endothelial growth factor
WST-1: Water-soluble tetrazolium 1.

## Supplementary Materials

Supplementary Materials Supplement Figure 1: influence of low-dose irradiation on metabolic activity of (A) EA.hy926 and (B) A549. The cells were irradiated with different doses; a WST-1 assay was performed at three time points after irradiation. The extinctions were normalized to samples of 0 Gy/2 hours. Error bars present the standard deviation (±SD) from four independent experiments; wells were assayed in triplicates in each of the experiments. Supplement Figure 2: influence of irradiation on cellular vitality of EA.hy926 cells. The cells were irradiated with different doses; the number of live and dead cells was counted at four time points after irradiation. Error bars present the standard deviation (±SD) from four independent experiments; wells were assayed in duplicates in each of the experiments; asterisks illustrate significance: ^∗^*p* < 0.05. Supplement Figure 3: accumulated levels of (A) E-selectin and (B) P-selectin in the supernatant of EA.hy926. The protein concentration was determined by a multiplex assay at five time points after irradiation with photons. Changes in protein concentrations are presented as mean (pg/mL) ± standard deviation (SD) from three independent experiments. Supplement Figure 4: accumulated levels of vascular endothelial growth factor (VEGF) in the supernatant of EA.hy926 endothelial cells. The protein concentration was determined by a multiplex assay at five time points after irradiation with photons. Changes in protein concentrations are presented as mean (pg/mL) ± standard deviation (SD) from three independent experiments; asterisks illustrate significance: ^∗^*p* < 0.05.
