# Supplementary material for: Effect of Ionizing Radiation on Human EA.hy926 Endothelial Cells under Inflammatory Conditions and Their Interactions with A549 Tumour Cells
Source: J Immunol Res. 2019 Sep 2;2019:9645481. doi: 10.1155/2019/9645481 (PMC6745109; doi:10.1155/2019/9645481)
Supplement: Supplementary Materials — Supplement Figure 1: influence of low-dose irradiation on metabolic activity of (A) EA.hy926 and (B) A549. The cells were irradiated with different doses; a WST-1 assay was performed at three time points after irradiation. The extinctions were normalized to samples of 0 Gy/2 hours. Error bars present the standard deviation (±SD) from four independent experiments; wells were assayed in triplicates in each of the experiments. Supplement Figure 2: influence of irradiation on cellular vitality of EA.hy926 cells. The cells were irradiated with different doses; the number of live and dead cells was counted at four time points after irradiation. Error bars present the standard deviation (±SD) from four independent experiments; wells were assayed in duplicates in each of the experiments; asterisks illustrate significance: ∗p < 0.05. Supplement Figure 3: accumulated levels of (A) E-selectin and (B) P-selectin in the supernatant of EA.hy926. The protein concentration was determined by a multiplex assay at five time points after irradiation with photons. Changes in protein concentrations are presented as mean (pg/mL) ± standard deviation (SD) from three independent experiments. Supplement Figure 4: accumulated levels of vascular endothelial growth factor (VEGF) in the supernatant of EA.hy926 endothelial cells. The protein concentration was determined by a multiplex assay at five time points after irradiation with photons. Changes in protein concentrations are presented as mean (pg/mL) ± standard deviation (SD) from three independent experiments; asterisks illustrate significance: ∗p < 0.05. [file 9645481.f1.docx]

**Supplement Figure 1**

| **Metabolic activity after irradiation** |
| --- |
| **A) EA.hy926** |
|  |
| **B) A549** |
|  |

**Supplement figure 1: Influence of low-dose irradiation on metabolic activity of A)** EA.hy926 and **B)** A549. The cells were irradiated with different doses; a WST-1 assay was performed at three time points after irradiation. The extinctions were normalized to samples of 0Gy/2hours. Error bars present the standard deviation (±SD) from four independent experiments; wells were assayed in triplicates in each of the experiments.

**Supplement Figure 2**

| **Measurement of live/dead cells after irradiation** |
| --- |
| 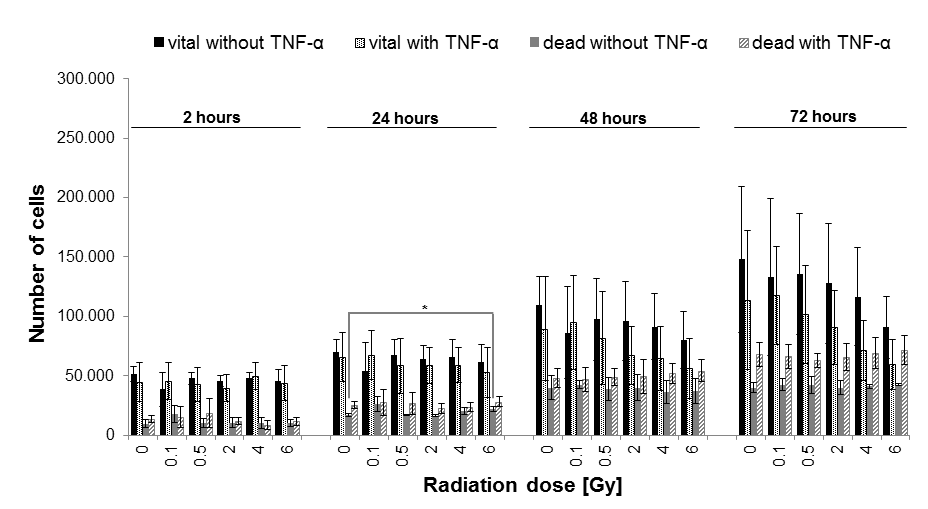 |
|  |

**Supplement figure 2: Influence of irradiation on cellular vitality of EA.hy926 cells**. The cells were irradiated with different doses; number of live and dead cells was counted at four time points after irradiation. Error bars present the standard deviation (±SD) from four independent experiments; wells were assayed in duplicates in each of the experiments; Asterisks illustrate significance: *p < 0.05.

**Supplement Figure 3**

| **Release of Selectins after irradiation** |
| --- |
| **A) E-Selectin**  **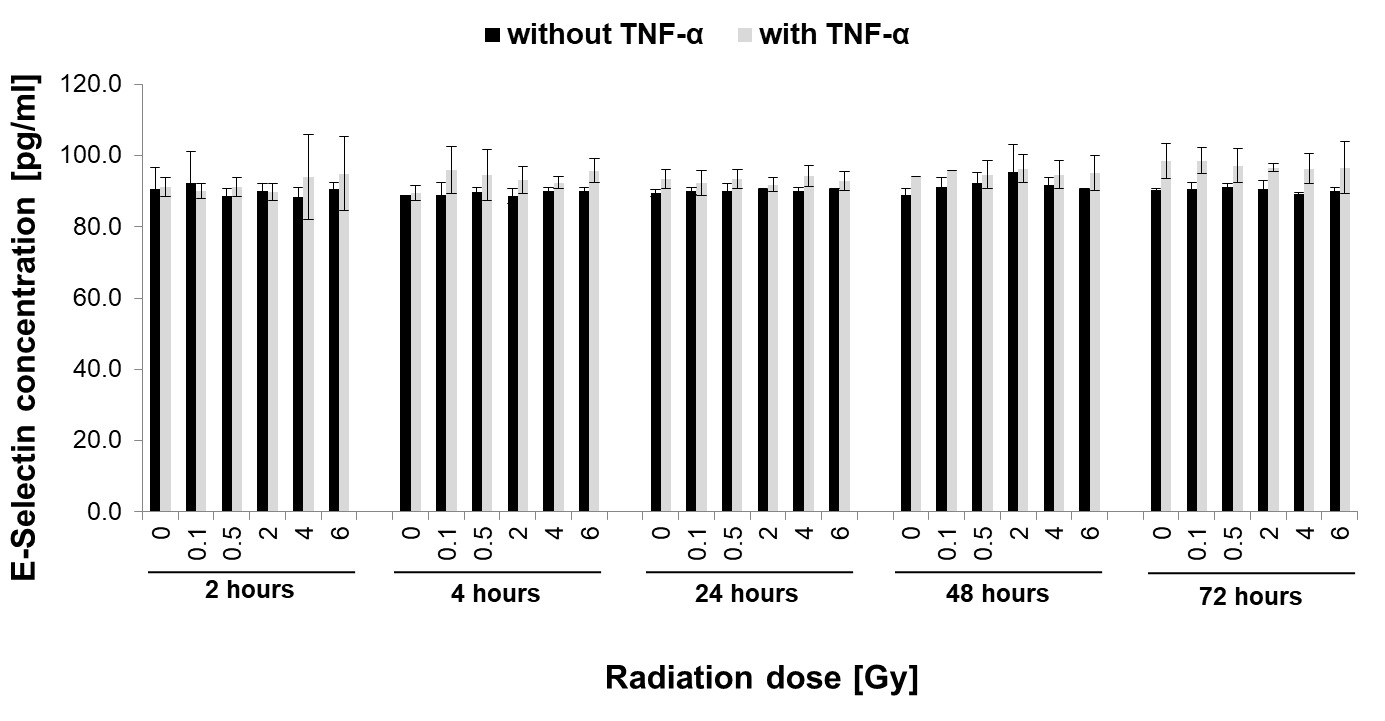** |
| **B) P-Selectin**  **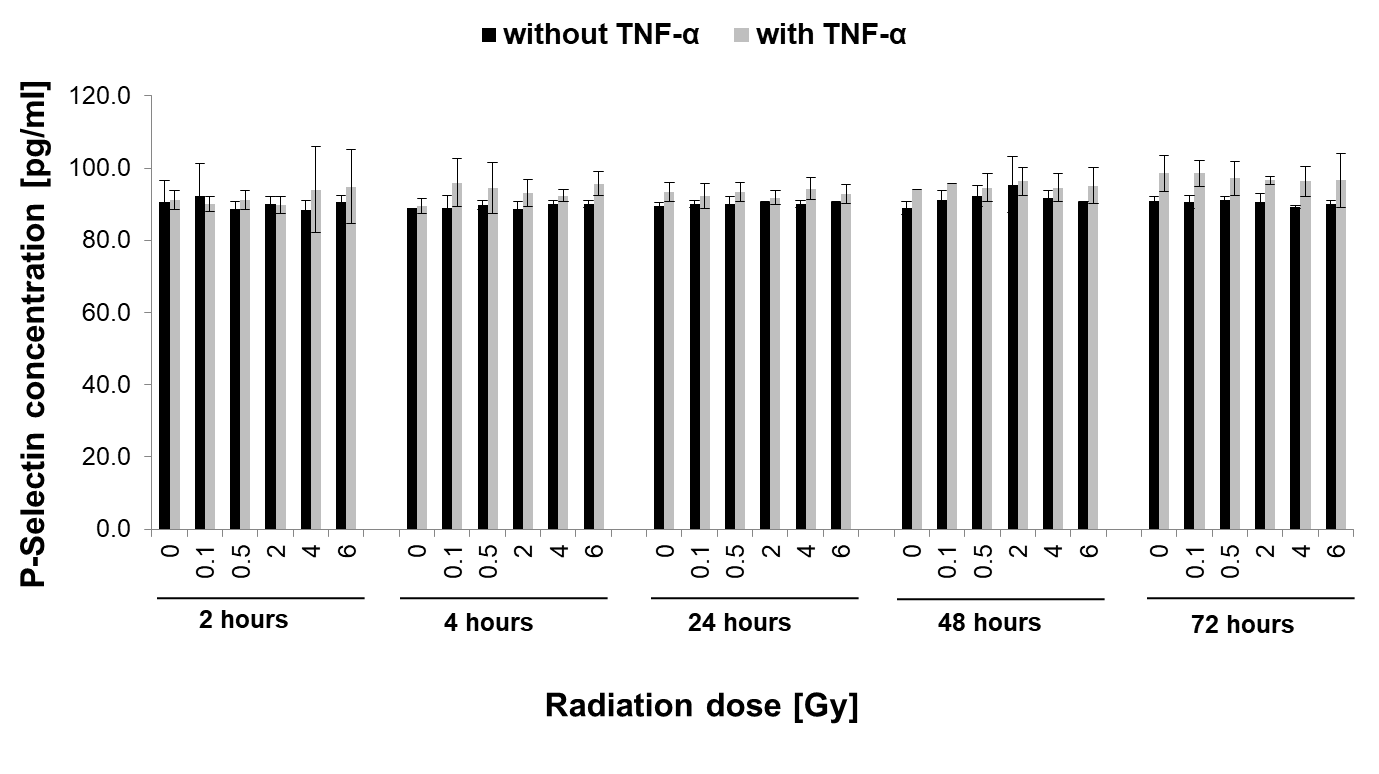** |

**Supplement figure 3: Accumulated levels of A)** E-Selectin and **B)** P-Selectin in the supernatant of EA.hy926. The protein concentration was determined by multiplex assay at five time points after irradiation with photons. Changes in protein concentrations are presented as mean (pg/ml) ± standard deviation (SD) from three independent experiments.

**Supplement Figure 4**

| **Release of VEGF after irradiation** |
| --- |
| **EA.hy926** |
|  |

**Supplement figure 4: Accumulated levels of Vascular Endothelial Growth Factor (VEGF) in the supernatant of EA.hy926 endothelial cells.** The protein concentration was determined by multiplex assay at five time points after irradiation with photons. Changes in protein concentrations are presented as mean (pg/ml) ± standard deviation (SD) from three independent experiments; Asterisks illustrate significance: *p < 0.05.
